# DIFFERENCES IN MEDICARE RESOURCE USE BY ENGLISH PROFICIENCY

**DOI:** 10.1093/geroni/igad104.1002

**Published:** 2023-12-21

**Authors:** Lilly Estenson, Eric Roberts, Mireille Jacobson

**Affiliations:** University of Southern California, Los Angeles, California, United States; University of Pennsylvania, Perelman School of Medicine, Philadelphia, Pennsylvania, United States; University of Southern California, Los Angeles, California, United States

## Abstract

Medicare is complicated and coverage decisions can be complex. Accordingly, the Centers for Medicare & Medicaid Services (CMS) provides a hotline, handbook, and website to help Medicare beneficiaries understand their benefits and coverage options. In this study, we used 2016-2018 Medicare Current Beneficiary Survey data and linear probability models to assess whether the likelihood of CMS Medicare resource use differed between beneficiaries with English proficiency and those with limited English proficiency (LEP). Our primary outcome was an indicator of having used at least one CMS resource. We considered English proficiency in both speaking and reading. Limited English-speaking proficiency or, separately, limited English-reading proficiency signified that the beneficiary reported speaking or reading English “not well” or “not at all.” Among a nationally representative sample of noninstitutionalized Medicare beneficiaries, 5.7% had limited English-speaking proficiency, 8.7% had limited English-reading proficiency, and 67.0% had used a CMS resource. Adjusting for health and social factors, age, language spoken at home, county-level Medicare Advantage characteristics, and state and year fixed effects, beneficiaries with limited English-reading proficiency were 16.7 percentage points less likely to have used a CMS resource compared to beneficiaries who reported reading English “well” or “very well” (p <.001). Limited English-speaking proficiency was not associated with CMS resource use. These findings have implications for federal initiatives to improve language access in Medicare, particularly efforts to reduce literacy barriers. Differences in Medicare coverage choice and benefits utilization among beneficiaries with LEP may be partially explained by differential access to or engagement with written CMS Medicare information.

